# Solid state fluorescence of proteins in high throughput mode and its applications

**DOI:** 10.12688/f1000research.2-82.v2

**Published:** 2019-03-25

**Authors:** Saurabh Gautam, Munishwar N Gupta

**Affiliations:** 1 Department of Chemistry, Indian Institute of Technology Delhi, New Delhi, 110016, India

**Keywords:** Chromophores, Emission spectra, Enzymes, Fluorescence resonance energy transfer, Green fluorescent protein, Lyophilization, Powders, Solutions

## Abstract

Direct comparison between fluorescence spectra of a sample in solution and solid state form is valuable to monitor the changes in protein structure when it is “dried” or immobilized on a solid surface (for biocatalysis or sensor applications). We describe here a simple method for recording fluorescence emission spectra of protein powders without using any dedicated accessory for solid samples in a high-throughput format. The 96-well plate used in our studies, was coated black from all the sides and the excitation and emission paths are identical and are from the top of the well. These two features minimize scatter and provide fairly noise free spectra. Even then the fluorescence intensity may be dependent upon many factors such as the extent of protein aggregation, morphology and sizes of the protein particles. Hence, (changes in) λ _max_ emission may be a more reliable metric in the case of fluorescence spectra of proteins in the solid state. However, any large changes in the intensity could indicate changes in the microenvironment of the fluorophore. The fluorescence emission spectra were blue-shifted (4 to 9 nm), showed an increase in the intensity for different proteins studied upon lyophilization, and were similar to what has been reported by others using available commercial accessories for solid state samples. After validating that our method worked just as well as the dedicated accessories, we applied the method to compare the fluorescence emission spectra of α-chymotrypsin in solution, precipitated form, and the lyophilized powder form. We further examined the fluorescence emission spectra of green fluorescent protein (GFP) in solution and solid form. We also analyzed fluorescence resonance energy transfer (FRET) between tryptophan (Trp57) and the cyclic chromophore of GFP. These findings pointed towards the change in the microenvironment around the cyclic chromophore in GFP upon lyophilization.

## Introduction

Fluorescence spectroscopy is a powerful tool to study protein structure ^1– 3^. Measurement of the fluorescence of proteins, when the latter is present in the solid state, is useful in several different contexts. Solid state fluorescence has a number of uses including in protein assays with protein electrophoresis samples ^4^, enzyme immobilization ^5^, microscopy ^6^, detecting changes in protein tertiary structure upon lyophilization ^7^, sensors and microarrays ^8, 9^and characterizing solid waste ^10^. Of these, fluorescence measurement of lyophilized samples is itself valuable for a variety of different kinds of studies. Enzyme catalysis in low water media is often carried out with lyophilized enzyme powders ^11– 14^. Only recently, circular dichroism (CD) of α-chymotrypsin “dried” (bulk water removed) with different methods has been reported with the help of a special accessory for recording CD spectra of solid samples as suspensions ^15^.

Some commercially available accessories for spectrofluorimeters allow recording the fluorescence emission spectra of the solid samples ^7, 16– 19^. These available commercial accessories can only accommodate solid samples and hence do not allow a direct comparison between fluorescence spectra of a sample in solution and solid state form. These accessories also do not allow working in a high throughput mode.

We describe here a simple method for recording fluorescence emission spectra of protein powders without using any dedicated accessory. This method works with a 96-well plate format. It enables the comparison of fluorescence spectra of a sample in a solid state with solution spectra, using comparable quantities of protein. It was found that, just like spectra recorded with these commercial accessories, the spectra of lyophilized powders obtained by our method showed a blue shift of λ _max_ (as compared to the solution spectra). After this validation, the method was used for two specific applications. In the first case, the method was used for assessing the tertiary structure of “dried” α-chymotrypsin. It was also used to track changes in fluorescence spectra of green fluorescent protein (GFP) when it is dried. While the former application is relevant to non-aqueous enzymology, the latter provides some insight into fluorescence resonance energy transfer (FRET) between tryptophan of GFP (Trp57) and its cyclic chromophore ^20, 21^.

These illustrative examples show that the valuable information provided by fluorescence emission spectroscopy about conformational changes in proteins upon drying can be obtained in a simple manner by anybody with a fluorescence-based microplate reader.

## Materials

Ampicillin, bovine serum albumin (BSA, cat. no. A7030), α-chymotrypsin (protease from bovine pancreas, cat. no. C4129), lysozyme (from chicken egg white, cat. No. L6876), phenylmethanesulfonylfluoride (PMSF) and n-propanol were purchased from Sigma-Aldrich (St. Louis, MO, USA). Isopropyl β-D-thiogalactopyranoside (IPTG) and LB medium were obtained from Himedia Laboratories (Mumbai, India). TLL ( *Thermomyces lanuginosus lipase*) was a kind gift from Novozymes (Denmark). *Candida rugosa* lipase was a gift from Amano Enzyme Inc. (Nagoya, Japan). Ninety-six well polystyrene microplates (Black, cat. no. 205003) were obtained from Porvair Sciences (Leatherhead, UK). All other chemicals used were of analytical grade. All the proteins used were >95% pure on SDS-PAGE.

## Overexpression and isolation of GFP

The plasmid pGFPuv expressing recombinant GFP was transformed into *E. coli* BL21(DE3) ^22^. A single colony was picked and inoculated into 5 mL LB medium containing 100 μg mL ^-1^ ampicillin. In total, 1% of primary inoculum was transferred into 1 L fresh LB broth (amp+) and grown at 37°C with shaking at 200 rpm until absorbance at 600 nm reached 0.8. Induction was carried out by adding 1 mM isopropyl β-D-thiogalactopyranoside (IPTG) (final concentration). The culture was further grown under similar conditions for 12 h. The cells were harvested by centrifugation at 8000 *g* for 10 min at 4°C. GFP was isolated from *E. coli* cells by sonication in 50 mM phosphate buffer, pH 7.5, containing 2 M NaCl and 100 μM phenylmethanesulfonylfluoride (PMSF), three times with 15 s pulses on ice, and centrifugation at 9000 *g* for 10 min at 4°C. The supernatant thus obtained was used as a crude extract for GFP and further purified to homogeneity (as shown by single band on SDS-PAGE) by immobilized metal affinity chromatography using nickel-alginate beads as described earlier ^22^.

## Lyophilization

Lyophilization of all the proteins was carried out on a freeze dryer from Allied Frost (New Delhi, India). Proteins were dialyzed against buffer (10 mM Tris-HCl, pH 7.0 for BSA, TLL, lysozyme, CRL and α-chymotrypsin; and 10 mM phosphate buffer, pH 7.5 for GFP) and were frozen at –70°C for 1 h before lyophilization.

## Preparation of enzyme precipitated and rinsed with propanol (EPRP) of α-chymotrypsin

Enzyme precipitated and rinsed with propanol (EPRP) of α-chymotrypsin was prepared as described previously ^15^. A total of 4 mg of α-chymotrypsin was dissolved in 400 μL of 10 mM Tris-HCl buffer, pH 7.8. Enzyme solution was then added drop wise to 4 ml of n-propanol with stirring at 4°C. After addition, the suspension was stirred for 30 min at 4°C. The suspension was then centrifuged at 5000 *g* for 10 min at 4°C, and the precipitate was rinsed three times with dry and chilled n-propanol.

## Fluorescence measurements

All fluorescence spectra were recorded on a Cary Eclipse, Varian spectrofluorimeter (Varian Inc., Mulgrave, Victoria, Australia) at 25°C by using the microtitre plate reader accessory for reading 96-well microplates. The typical protein concentration of proteins used for fluorescence measurements in solution was 2 mg/mL in a total volume of 200 μL. Proteins were lyophilized at the same concentration and same amount of protein was used for solid state fluorescence measurements. The fluorescence emission spectra were recorded from 300 nm to 400 nm upon excitation at 280 nm ^2^. For GFP, the fluorescence emission spectra were recorded from 450 nm to 600 nm upon excitation at 395 nm ^23^. The excitation and emission slit widths were kept at 2 nm and 5 nm, respectively. All fluorescence spectra were normalized and corrected for background contributions including the buffer.

## Estimation of protein concentration

Protein concentration was estimated by the dye binding method using bovine serum albumin as the standard protein ^24^.

## Results and discussion

**Figure 1.**
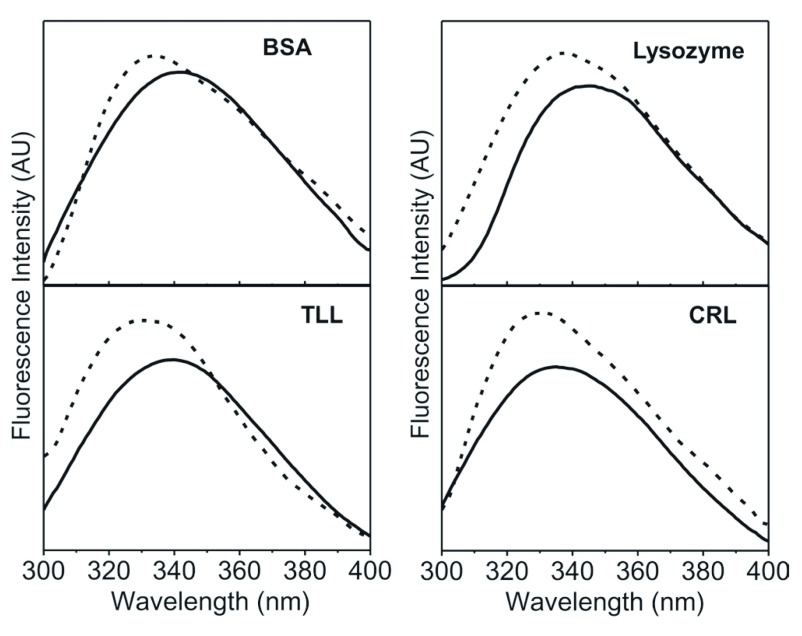
Fluorescence emission spectra (AU, arbitrary units) of four different proteins [Bovine serum albumin (BSA), *Thermomyces lanuginosus* lipase (TLL), *Candida rugosa* lipase (CRL)]. Protein in aqueous buffer (10 mM Tris-HCl, pH 7.0) (—) and lyophilized protein powder (- -). All these fluorescence emission spectra were recorded with excitation at 280 nm using excitation and emission slit widths of 2 nm and 5 nm, respectively.

The method developed here consists of simply placing the lyophilized powder of the protein in the well of 96-well microplate. The fluorescence spectra were recorded on a standard Varian microplate reader. The 96- well plates used were black from all sides. The emission in this set up takes place along with the same path direction as the exciting radiation and this is from top of the wells. This minimizes scattered light and distortion of the spectra. This results in the spectra which do not show much noise as seen in the raw data given in the data files. The λ _max_ excitation known for the protein solutions were used for solid samples as well. Intrinsic fluorescence emission spectra of four different commercial proteins were obtained after lyophilization from the aqueous buffer and compared with the spectra of the respective protein in the aqueous buffer solution ( Figure 1). The amount of protein in each solution was the same as was used for obtaining the lyophilized powders. In all the cases there was a blue shift in emission λ _max_ ( Table 1) and an increase in the intensity of the emission spectra of the lyophilized proteins as compared to the protein in aqueous solution. The intensity, of course, would be dependent upon many factors such as extent of protein aggregation, morphology and sizes of the protein particles. Nevertheless, it is worth noting that a similar blue shift as well as increase in the intensity have been reported by Ramachander *et al.* ^7^ while comparing the solid state and solution state fluorescence spectra of four therapeutic proteins (the identities of the proteins were not disclosed by these authors). These workers had used a special accessory (called a solid state holder set up) for the Cary Eclipse spectrofluorimeter. The blue shift in the lyophilized state reflects that the environment of intrinsic fluorophores is more non-polar. This is expected due to the removal of water. The small differences in the extent of the blue shift ( Table 1) in case of the four proteins presumably originate from the differences in the microenvironments of tryptophan in the folded structure of each of the proteins. To start with, when in solution, the microenvironments of tryptophan are expected to be different between different proteins.

Fluorescence emission spectra of Bovine serum albumin (BSA), Thermomyces lanuginosus lipase (TLL), Candida rugosa lipase (CRL). Figure 1. Fluorescence emission spectra (AU, arbitrary units) of four different proteins [Bovine serum albumin (BSA), Thermomyces lanuginosus lipase (TLL), Candida rugosa lipase (CRL)]. Data for proteins in aqueous buffer (10 mM Tris-HCl, pH 7.0) and lyophilized protein powders are given. All these fluorescence emission spectra were recorded with excitation at 280 nm using excitation and emission slit widths of 2 nm and 5 nm, respectively. Click here for additional data file. Copyright: © 2019 Gautam S and Gupta MN 2019 Data associated with the article are available under the terms of the Creative Commons Zero "No rights reserved" data waiver (CC0 1.0 Public domain dedication).

**Table 1.** Fluorescence emission λ _max_ (nm) of four different lyophilized proteins and their comparison with the proteins in 10 mM Tris-HCl, pH 7.0.

Protein	λ _max_ (nm) of the protein in aqueous buffer solution	λ _max_ (nm) of the lyophilized protein	Change in λ _max_ (nm)
BSA ^a^	341±1	334±1	–7
TLL ^b^	340±1	332±1	–8
Lysozyme	345±1	338±1	–7
CRL ^c^	334±1	330±1	–4

^a^BSA = Bovine serum albumin, ^b^TLL = *Thermomyces lanuginosus* lipase, ^c^CRL = *Candida rugosa* lipase.

**Figure 2.**
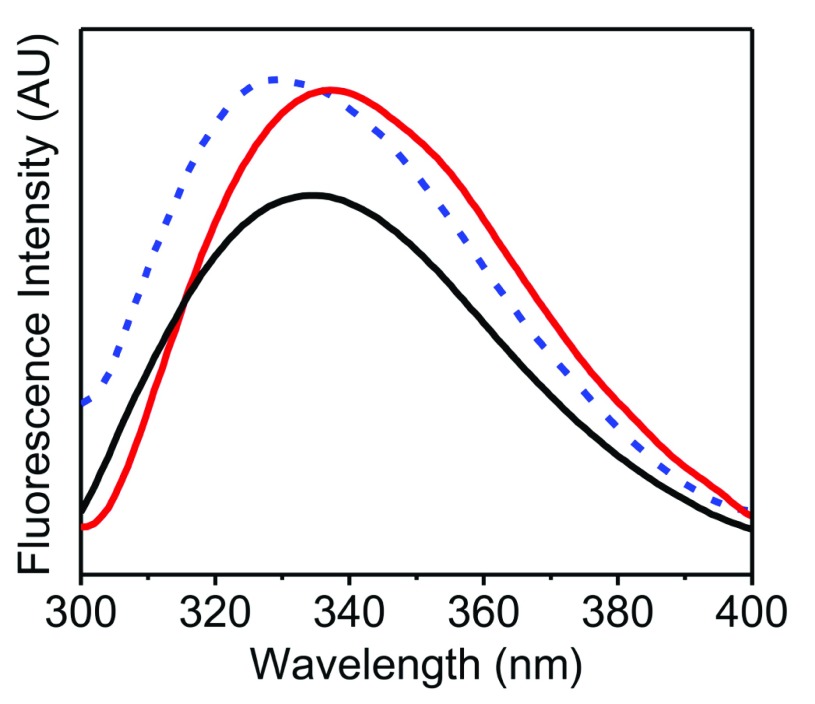
Fluorescence emission spectra (AU, arbitrary units) of α-chymotrypsin. α-Chymotrypsin in aqueous buffer (10 mM Tris-HCl, pH 7.0) (black curve), lyophilized α-chymotrypsin powder (blue dashed curve) and solid sample of enzyme precipitated and rinsed with propanol (EPRP) of α-chymotrypsin (red curve). All these fluorescence emission spectra were recorded with excitation at 280 nm using excitation and emission slit widths of 2 nm and 5 nm, respectively.

Figure 2 shows the fluorescence emission spectra of α-chymotrypsin in solution and in the solid state. Native α-chymotrypsin in aqueous buffer showed emission λ _max_ of 335 nm while upon lyophilization it was blue shifted to 328 nm with an increase in the intensity. This is likely again due to the non-polar environment of the aromatic residues. It has been shown that lyophilized preparations of α-chymotrypsin show poor esterification/transesterification activity in low water media containing organic solvents ^25^. Low activities of lyophilized powders in such media have been explained due to structural changes which occur upon lyophilization ^14^. “Dry” preparations obtained by precipitation of α-chymotrypsin from its aqueous solution by addition of water miscible organic solvents are known to show much better activities in low water media ^15, 26, 27^. Recently, Solanki et al. ^15^ found that changes in the CD spectra upon “drying” correlated well with catalytic activities in low water media for various α-chymotrypsin preparations. A high activity preparation of α-chymotrypsin for low water media (EPRP) ^15^ showed a very small red shift in the emission λ _max_ to 337 nm with an increase in the intensity of the emission spectra, in contrast to the lyophilized protein which showed a blue shift. This further highlights that the lyophilized protein is different from the high activity preparation (EPRP) in terms of the tertiary structure, demonstrating that the simple fluorescence method proposed here can successfully monitor changes in the tertiary structure of different types of formulations of solid proteins.

Fluorescence emission spectra of α-chymotrypsin. Figure 2. Fluorescence emission spectra (AU, arbitrary units) of α-chymotrypsin. α-Chymotrypsin in aqueous buffer (10 mM Tris-HCl, pH 7.0), lyophilized α-chymotrypsin powder and solid sample of enzyme precipitated and rinsed with propanol (EPRP) of α-chymotrypsin. All these fluorescence emission spectra were recorded with excitation at 280 nm using excitation and emission slit widths of 2 nm and 5 nm, respectively. Click here for additional data file. Copyright: © 2019 Gautam S and Gupta MN 2019 Data associated with the article are available under the terms of the Creative Commons Zero "No rights reserved" data waiver (CC0 1.0 Public domain dedication).

**Figure 3.**
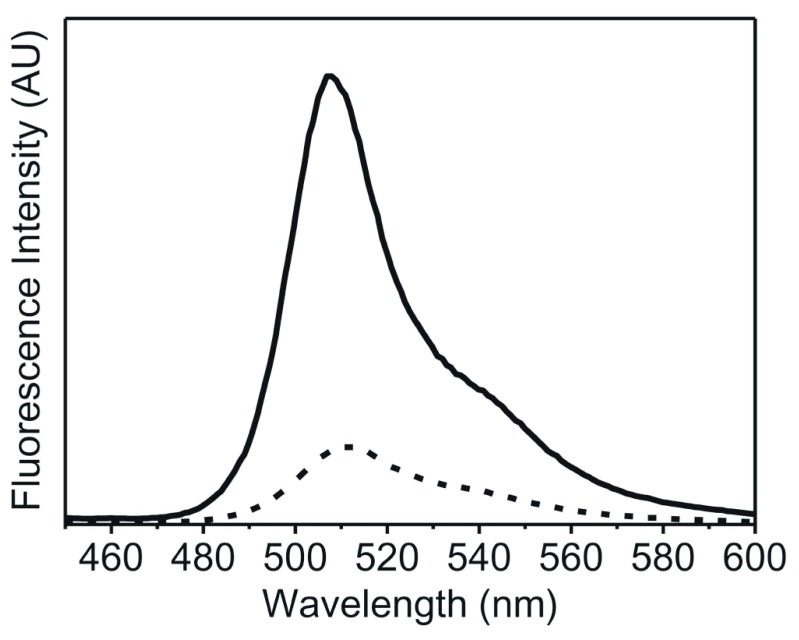
Fluorescence emission spectra (AU, arbitrary units) of green fluorescent protein (GFP). GFP in aqueous buffer (50 mM PBS) (solid line) and lyophilized powder of GFP (dashed line). These fluorescence emission spectra were recorded with excitation at 395 nm using excitation and emission slit widths of 2 nm and 5 nm, respectively.

**Figure 4.**
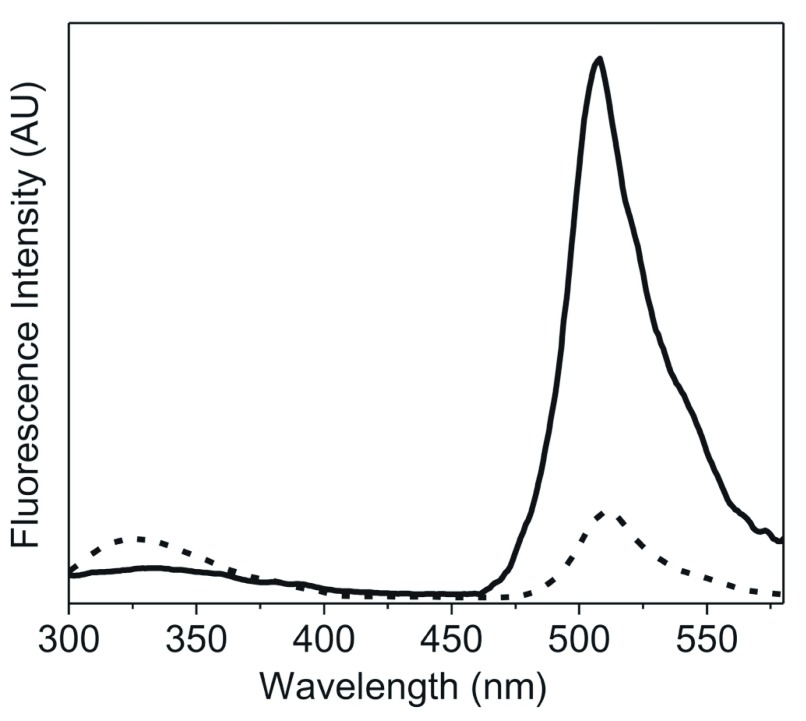
Fluorescence emission spectra (AU, arbitrary units) of green fluorescent protein (GFP) showing fluorescence resonance energy transfer (FRET) between tryptophan (Trp57) and cyclic chromophore. GFP in aqueous buffer (50 mM PBS) (solid line) and lyophilized powder of GFP (dashed line). These fluorescence emission spectra were recorded with excitation at 295 nm using excitation and emission slit widths of 2 nm and 5 nm, respectively.

To further examine the application of this new method, we recorded the fluorescence spectra of the lyophilized formulation of recombinant GFP. In this case, the high intrinsic fluorescence of GFP due to the cyclic moiety present in the protein, which is very sensitive to changes in the structure of protein ^23^, was studied ( Figure 3). The lyophilized protein showed a considerable decrease in the intensity of the fluorescence emission spectra to 17% as compared to that of GFP in solution. As pointed out earlier, changes in the fluorescence intensity could arise from various parameters. However, in this case the decrease in the fluorescence intensity was very large. The emission λ _max_ was also slightly red shifted upon lyophilization (4 nm, from 508 nm to 512 nm). These changes (in fluorescence intensity and shift of λ _max_ emission) were opposite to what was observed with other proteins ( Figure 1). Visser *et al.* ^20^ have shown that the fluorescence of the cyclic chromophore in GFP results from the energy transfer from the tryptophan. Figure 4 shows that the energy transfer between the tryptophan and the cyclic chromophore is much less in the lyophilized form. It is noteworthy that the change in the emission intensity due to tryptophan residues (at ~340 nm) was observed in GFP ( Figure 4), just as for the other proteins ( Figure 1). Both changes reflect how the microenvironment affects the emission fluorescence of the unique chromophore of GFP and could be due to the degradation of this cyclic chromophore upon lyophilization.

So, while a shift in the λ _max_ of emission is the reliable metric to look at while comparing the solid state spectra with solution spectra, in a case like GFP where large changes in intensity is observed, it should not be ignored as it could indicate drastic changes in the microenvironment of the fluorophore.

Fluorescence emission spectra of green fluorescent protein (GFP). Figure 3. Fluorescence emission spectra (AU, arbitrary units) of green fluorescent protein (GFP). GFP in aqueous buffer (50 mM PBS) and lyophilized powder of GFP. These fluorescence emission spectra were recorded with excitation at 395 nm using excitation and emission slit widths of 2 nm and 5 nm, respectively. Click here for additional data file. Copyright: © 2019 Gautam S and Gupta MN 2019 Data associated with the article are available under the terms of the Creative Commons Zero "No rights reserved" data waiver (CC0 1.0 Public domain dedication).

Fluorescence emission spectra of green fluorescent protein (GFP) showing fluorescence resonance energy transfer (FRET) between tryptophan (Trp57) and cyclic chromophore. Figure 4. Fluorescence emission spectra (AU, arbitrary units) of green fluorescent protein (GFP) showing fluorescence resonance energy transfer (FRET) between tryptophan (Trp57) and cyclic chromophore. GFP in aqueous buffer (50 mM PBS) and lyophilized powder of GFP. These fluorescence emission spectra were recorded with excitation at 295 nm using excitation and emission slit widths of 2 nm and 5 nm, respectively. Click here for additional data file. Copyright: © 2019 Gautam S and Gupta MN 2019 Data associated with the article are available under the terms of the Creative Commons Zero "No rights reserved" data waiver (CC0 1.0 Public domain dedication).

## Data availability

The data referenced by this article are under copyright with the following copyright statement: Copyright: © 2019 Gautam S and Gupta MN

Data associated with the article are available under the terms of the Creative Commons Zero "No rights reserved" data waiver (CC0 1.0 Public domain dedication). 

*Figshare*: Fluorescence emission spectra of Bovine serum albumin (BSA), Thermomyces lanuginosus lipase (TLL), Candida rugosa lipase (CRL). doi: https://doi.org/10.6084/m9.figshare.640095.v3 ^28^

*Figshare*: Fluorescence emission spectra of α-chymotrypsin. doi: https://doi.org/10.6084/m9.figshare.640096.v3 ^29^

*Figshare*: Fluorescence emission spectra of green fluorescent protein (GFP). doi: https://doi.org/10.6084/m9.figshare.640097.v3 ^30^

*Figshare*: Fluorescence emission spectra of green fluorescent protein (GFP) showing fluorescence resonance energy transfer (FRET) between tryptophan (Trp57) and cyclic chromophore. doi: https://doi.org/10.6084/m9.figshare.640098.v3 ^31^

## Conclusion

A simple method of placing the dry protein powder in a 96-well microplate enables the generation of fluorescence spectra of a protein in the solid state. As the fluorescence spectra of the solution can also be recorded in an identical fashion, the exact comparison between the solution and solid state spectra is possible.

## Author contributions

MNG designed the study. MNG and SG participated in the interpretation of data and the writing of the manuscript. SG carried out the experimental work. Both authors approved the submission of the final manuscript.
