# Supplementary material for: Solid state fluorescence of proteins in high throughput mode and its applications
Source: F1000Res. 2019 Mar 25;2:82. Originally published 2013 Mar 11. [Version 2] doi: 10.12688/f1000research.2-82.v2 (PMC6441877; doi:10.12688/f1000research.2-82.v2)
Supplement: Peer review history

# Peer review: Reviewer response for version 2

**DOI:** 10.5256/f1000research.20258.r46209

**Published:** 2019-03-26

**Authors:** Vladimir Uversky

**Affiliations:** 1 Department of Molecular Medicine, Department of Molecular Medicine, University of South Florida, Tampa, FL, USA

The authors adequately addressed my critical points and introduced the necessary changes to the manuscript. In my view, this amended version is a useful study that will have a noticeable impact. I generally agree with the statement of the authors that the large changes in the fluorescence intensity could reflect some changes in the fluorophore microenvironment. However, keeping in mind extreme sensitivity of fluorescence intensity to various factors, I would also strongly recommend to use both fluorescence intensity and λ _max_ emission as the most reliable metrix in this case.

I have read this submission. I believe that I have an appropriate level of expertise to confirm that it is of an acceptable scientific standard.

# Peer review: Reviewer response for version 2

**DOI:** 10.5256/f1000research.20258.r46208

**Published:** 2019-03-26

**Authors:** Vincent Rotello

**Affiliations:** 1 Department of Chemistry, University of Massachusetts Amherst, Amherst, MA, USA

The manuscript looks good to me.

I have read this submission. I believe that I have an appropriate level of expertise to confirm that it is of an acceptable scientific standard.

# Peer review: Reviewer response for version 1

**DOI:** 10.5256/f1000research.872.r838

**Published:** 2013-03-15

**Authors:** Vladimir Uversky

**Affiliations:** 1 Department of Molecular Medicine, Department of Molecular Medicine, University of South Florida, Tampa, FL, USA

The suggested technique for the analysis of solid state fluorescence of proteins is simple and can find multiple applications. Although the authors analyzed both fluorescence intensity and λ _max_ of protein powders and corresponding protein solutions, the applicability of fluorescence intensity is questionable. In fact, fluorescence intensity depends on a wide range of factors and cannot be easily interpreted, especially if samples are in different aggregated states (solid versus solution). Therefore, only λ _max_ should be taken as a parameter for analysis since this characteristic has understandable physical grounds.

I have read this submission. I believe that I have an appropriate level of expertise to confirm that it is of an acceptable scientific standard, however I have significant reservations, as outlined above.

# Response

**Published:** 2013-03-15

**Authors:** Munishwar N Gupta

**Affiliations:** Indian Institute of Technology Delhi, India

The references provided in the manuscript to the earlier work carried out with commercial accessories described differences between the solution spectra and the solid state spectra and list changes in both intensity and λ _max_ shift. In order to validate our method, we wanted to point out that both/similar changes occur with our method as well. However, as Table 1 of our manuscript shows, we have focused on the peak position. The amended manuscript can point out that intensities can be affected by many variables and one should rely more upon the λ _max_ position. These explanations can be added in the amended manuscript.

# Peer review: Reviewer response for version 1

**DOI:** 10.5256/f1000research.872.r834

**Published:** 2013-03-13

**Authors:** Vincent Rotello

**Affiliations:** 1 Department of Chemistry, University of Massachusetts Amherst, Amherst, MA, USA

The use of solid state fluorescence to provide protein quality control is a modest technical advance. This study looks at both fluorescence peak position and intensity. The latter value, however, will be affected by light scattering that is both particle size and morphology dependent. I would suggest focusing on the peak position as this will provide a much better metric.

I have read this submission. I believe that I have an appropriate level of expertise to confirm that it is of an acceptable scientific standard, however I have significant reservations, as outlined above.

# Response

**Published:** 2013-03-15

**Authors:** Munishwar N Gupta

**Affiliations:** Indian Institute of Technology Delhi, India

1. The work is not aimed at suggesting that fluorescence emission spectroscopy should be applied to solid samples of proteins instead of using their solutions. On the other hand, as discussed in the manuscript, there are many investigations (enzyme immobilization on solid surfaces, enzyme powders in low water enzymology) wherein structural characterization would be valuable with the solid state samples. The availability of some commercial accessories fulfills this need. So, we offer a way of measuring fluorescence spectra in a 96-well format without any additional accessories.

2. The 96-well plates used were black from all sides except from the top. So, the emission takes place along the same path direction as the exciting radiation. This minimizes scattered light and distortion of the spectra. This results in the spectra which are of reasonable quality as seen in our raw data. 

3. The references provided in the manuscript to the earlier work carried out with commercial accessories described differences between the solution spectra and the solid state spectra and list changes in both intensity and λmax shift. In order to validate our method, we wanted to point out that both/similar changes occur with our method as well. However, as Table 1 of our manuscript shows, we have focused on the peak position. In the amended manuscript we will point out that intensities can be affected by many variables and that one should rely more upon the λmax position. These explanations will be added to our amended manuscript.
